# Tissue remodeling: a mating-induced differentiation program for the *Drosophila *oviduct

**DOI:** 10.1186/1471-213X-8-114

**Published:** 2008-12-08

**Authors:** Anat Kapelnikov, Patricia K Rivlin, Ronald R Hoy, Yael Heifetz

**Affiliations:** 1 Department of Entomology, The Hebrew University, Rehovot, Israel; 2 Department of Neurobiology and Behaviour, Cornell University, Ithaca, New York, USA

## Abstract

**Background:**

In both vertebrates and invertebrates, the oviduct is an epithelial tube surrounded by visceral muscles that serves as a conduit for gamete transport between the ovary and uterus. While *Drosophila *is a model system for tubular organ development, few studies have addressed the development of the fly's oviduct. Recent studies in *Drosophila *have identified mating-responsive genes and proteins whose levels in the oviduct are altered by mating. Since many of these molecules (e.g. Muscle LIM protein 84B, Coracle, Neuroglian) have known roles in the differentiation of muscle and epithelia of other organs, mating may trigger similar differentiation events in the oviduct. This led us to hypothesize that mating mediates the last stages of oviduct differentiation in which organ-specific specializations arise.

**Results:**

Using electron- and confocal-microscopy we identified tissue-wide post-mating changes in the oviduct including differentiation of cellular junctions, remodeling of extracellular matrix, increased myofibril formation, and increased innervation. Analysis of once- and twice-mated females reveals that some mating-responsive proteins respond only to the first mating, while others respond to both matings.

**Conclusion:**

We uncovered ultrastructural changes in the mated oviduct that are consistent with the roles that mating-responsive proteins play in muscle and epithelial differentiation elsewhere. This suggests that mating triggers the late differentiation of the oviduct. Furthermore, we suggest that mating-responsive proteins that respond only to the first mating are involved in the final maturation of the oviduct while proteins that remain responsive to later matings are also involved in maintenance and ongoing function of the oviduct. Taken together, our results establish the oviduct as an attractive system to address mechanisms that regulate the late stages of differentiation and maintenance of a tubular organ.

## Background

Most internal organs, including the vascular and respiratory systems and the gastro-intestinal and urinary-genital tracts are comprised of a single epithelial tube or a network of tubes. Tubular organs serve as conduits for the transport of gases, liquids, or solutes, and serve as barriers between biological compartments. To create tubes with specific flow and barrier properties, the morphology of the tube must be precisely specified during development and modulated by physiology. To accommodate specific physiological roles, tissue-specific programs for differentiation are employed at the last stages of development. While much is known about the molecular and cellular basis of tube formation [1-6], little is known about the mechanisms that regulate the late stages of differentiation in which organ-specific specializations arise.

The conservation of genes and similarity in tubular organ design across taxa make *Drosophila *an excellent model for understanding organogenesis in higher animals. In *Drosophila*, the best understood tubular organs from a developmental point of view are the trachea and salivary gland. Studies of these organs reveal a general program for tubular organ development, in which combinatorial expression of global patterning genes specifies positions within the embryo for the subsequent activation of tissue-specific early genes and transcription factors. This program results in the activation of downstream genes involved in terminal differentiation of organ-specific specializations such as the cuticle that lines the tracheal lumen [1,2,4,7-9].

The *Drosophila *female reproductive tract is another tubular system, consisting of the uterus and a common oviduct (the main tube) that branches into two lateral oviducts. Regional differences in function are observed along the length of the tract, with egg activation occurring largely at the proximal end, in the lateral oviducts, and fertilization occurring at the distal end, in the uterus [10,11]. Unlike other tubular organs, little is known about the development of the female reproductive tract. However, regional differences in function suggest the presence of region-specific differentiation programs within the female reproductive tract.

In *Drosophila*, mating induces changes in female behavior and physiology via molecules transmitted in the seminal fluid. These changes are rapid and lead to a mated female state which is profoundly different from the unmated female state. While an unmated female lays few eggs and readily accepts the courtship efforts of a male, a mated female exhibits increased egg-laying and actively rejects males [12-19]. Microarray studies of whole flies reveal that the changes in egg-laying rate are accompanied by a change in gene expression. Within three hours of mating there is an increase in expression of a small number of genes [20,21]. Rapid changes in gene expression, as well as protein abundance, have also been observed in the female reproductive tract [22,23].

In the upper reproductive tract (lateral and common oviducts, hereafter, oviduct), mating induces an increase in immune related transcripts and down regulates transcription factors involved in cell growth and differentiation. At the protein level mating induces increased abundance of proteins associated with muscle assembly and function and cytoskeletal proteins associated with epithelial morphogenesis [23]. Since many of these mating-responsive proteins act in late differentiation pathways of muscle and epithelia elsewhere (e.g. Bent, Muscle LIM protein 84B(Mlp84B), Neuroglian (Nrg), Coracle (Cora)), we hypothesize that mating triggers similar differentiation in the oviduct. To test our hypothesis we characterized the ultrastructure of oviduct epithelia and muscle, as well as the pattern of innervation before and after mating. We then examined the effect of different mating regimes on oviduct mating-responsive cytoskeletal proteins and on female reproductive output. Our results suggest that active tissue remodeling takes place in the oviduct epithelia and musculature in response to mating. Furthermore, we found a striking increase in innervation of the oviduct after mating.

Our results show that the reproductive tract is an attractive system to address mechanisms that regulate the late stages of tissue differentiation in a tubular organ. Unlike other tubular organs, the last differentiation stage of the oviduct is triggered by an extrinsic cue (mating). This makes it possible to experimentally control the onset of differentiation, with an opportunity to independently examine the effects of mating and age. In addition, it allows us to examine processes essential for reproduction.

## Results

### Mating induces changes in oviduct lumen

Our previous molecular profiling showed that mating promotes changes in actin-based cytoskeletal molecules and suggests that mating triggers molecular changes and tissue remodeling in the female reproductive tract that mediate its progression to a mature functional stage [23]. To gain insight into the mechanisms that underlie this progression, we used light and electron microscopy to determine the morphological status of the oviduct in unmated and mated 3-day old females.

**Figure 1:**
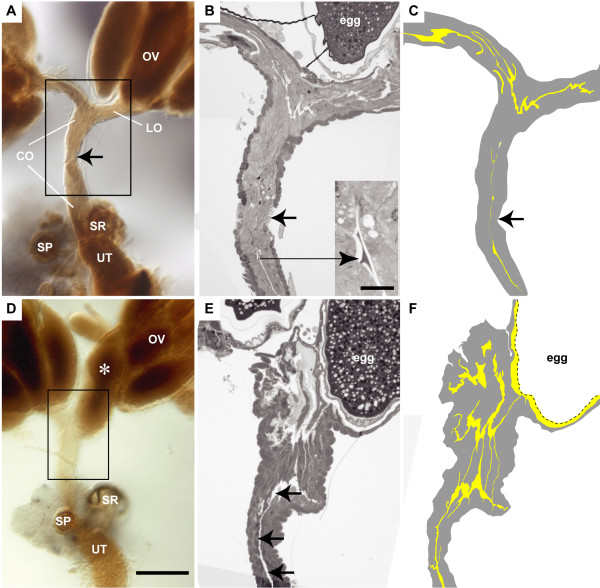
**Mating induces opening of the oviduct lumen**. (A-C) Unmated; (D-F) Mated; (A and D) Entire reproductive tract was embedded in plastic; ovary (OV), lateral oviducts (LO), common oviduct (CO), seminal receptacle (SR), spermatheca (SP), uterus (UT) are labeled. Scalebar is 200 μm. (C and F) Illustrations that highlight the lumenal space (yellow) are shown. (B and E) Toluidine blue stained 1-μm thick section of region marked by box in A and D, respectively. (A) Common oviduct appears constricted (arrow). (B and C) Lumen is visible in lateral oviducts, but is barely detectable in constricted region of oviduct (arrow). In the lower common oviduct, darkly stained material is observed in the lumen (inset; bar is 10 μm). (D) Egg fills one of the lateral oviducts. Common oviduct does not appear constricted (boxed region). (E and F) Oviduct lumen is more visible in mated females (arrows). Dark staining material is not detected in lumen.

In nearly all mated reproductive tracts processed for microscopy (8/9), an egg was located in one of the lateral oviducts, whereas an egg was never observed in the oviduct of unmated reproductive tracts (5/5) (Figure 1). This observation is consistent with previous studies that report increased ovulation and egg-laying at 6 h post-mating [24]. In all unmated reproductive tracts examined, the region between the lateral oviducts and the middle of the common oviduct was either tapered or constricted, whereas this region appeared relaxed in the mated reproductive tract (Figure 1A and 1D). These observations raise the possibility that the lumen is narrow in the unmated oviduct and larger in the mated oviduct. To address this possibility, we collected serial 1 μm longitudinal sections through the reproductive tracts of unmated and mated females and stained these sections with toluidine blue to survey the appearance of the lumen along the entire length of the oviduct (Figure 1B and 1E). Our examination reveals that, in unmated reproductive tracts, the lateral oviduct lumen has an irregular shape, while the common oviduct lumen appears straight. Moreover, in all the unmated reproductive tracts sectioned, we detected patches of darkly stained material in the lumen of the lower common oviduct. In the mated reproductive tract, the lumen of the upper oviduct (defined as the lateral oviduct and upper part of the common oviduct) has an irregular shape, and the lumen of the lower oviduct (defined as the lower part of common oviduct) appears straight. In addition, the lumen of the lower oviduct appears wider in mated than unmated reproductive tracts (Figure 1C and 1F). Interestingly, darkly stained material was not detected in the oviduct lumen of mated females. This observation appears to be consistent with the description made by Mahowald et al. [25], who reported that the oviduct lumen of unmated females is nearly filled with an intima-like matrix and that this matrix is reduced after mating.

Because 3-day-old mated females lay eggs, it is unclear whether the lack of lumenal material and increase in lumen size in mated females occurred before or after the passage of eggs. We suggest that mating directly or indirectly induces morphological changes in the oviduct that facilitate egg passage through the duct. Taken together, our observations lead us to propose that the oviduct lumen is closed and/or obstructed in the unmated reproductive tract, and that mating induces changes in the epithelia and/or muscle that "open" the oviduct lumen.

### Initial formation of cell-cell junctions in oviduct epithelia is mating-independent

**Figure 2:**
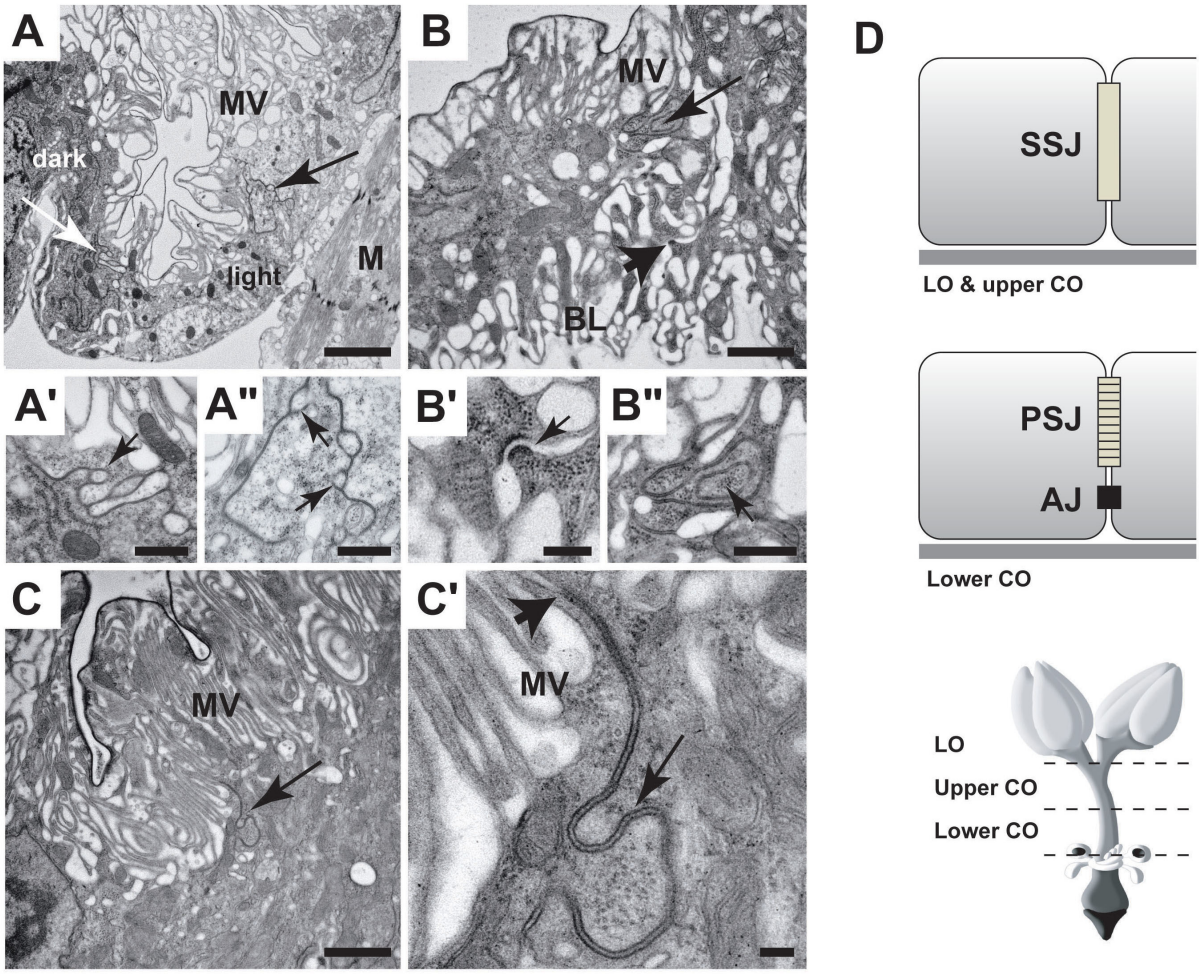
**Oviduct epithelia display region-specific apical morphology and septate junctions are formed prior to mating**. (A) *Upper oviduct (unmated)*: This is the only region where two types of epithelia cells (light and dark) are observed. Both cell types display apical microvilli (MV) and are joined by an extensive septate junction (SJ; white and black arrows); Muscle (M). Higher magnification of SJ between light and dark cells (A') and SJ between two light cells (A"). Note that both SJs are covered by interdigitations (arrow) and septae are not visible, but intercellular space is filled with electron dense material. (B) *Lower oviduct (mated)*: epithelia cells are joined apically by SJ (long arrow). Basolateral membrane (BL) gives rise to a labyrinth-like membrane which is joined laterally by spot adherens junctions (AJ; thick arrow). (B') Higher magnification of AJ; intercellular space (arrow) is filled with a filamentous material. (B") Higher magnification of SJ which is covered by interdigitations (arrow). (C) *Lower oviduct (unmated)*: Apical membrane gives rise to brush border-like microvilli (MV); epithelia cells are joined laterally by a SJ (arrow). (C') Higher magnification of SJ. Note the apical position of SJ (arrowhead) and ladder-like arrangement of septae (arrow). (D) Schematic summary of cell-cell junctions: Different types of junctions are observed in different regions of oviduct, but no differences are observed between unmated and mated females. SSJ (smooth-like septate junction), PSJ (pleated septate junction), AJ (adherens junction), LO (lateral oviduct), CO (common oviduct). Bar equals: A (2 μm), A' and A" (500 nm), B (1 μm), B' (200 nm), B" (500 nm), C (1 μm), C' (100 nm).

To determine whether mating induces specific morphological changes in the oviduct epithelia post-mating, we next examined the ultrastructure of the oviduct epithelia in unmated and mated reproductive tracts. Since molecular profiling demonstrates that proteins associated with cellular junctions such as α- and β-Spectrin (Spec), Cora, and Nrg [23] increase post-mating, we first determined the status of the cellular junctions in the oviduct epithelia of unmated females, and whether these junctions change post-mating. In *Drosophila*, most ectodermally derived epithelia (such as the epidermis and trachea), with a few exceptions, are joined apically by a belt-like adherens junction called the zonal adherens junction (ZA) followed basally by a septate junction (SJ) [26]. Our analysis reveals that the oviduct is lined, along its entire length, by a monolayered epithelium comprised of squamous-type cells. Although region-specific differences in morphology were observed, all oviduct epithelia examined, in both unmated and mated females, are joined along their lateral membranes by an extensive SJ and lack an apical ZA (Figure 2). SJs and ZAs form complete belts that surround the epithelial cell, thus making these junctions easily visible in transverse sections through the epithelium. Because ZAs were not detected in our transverse sections through the oviduct, this implies that ZAs never formed, or developed earlier and were lost (Figure 2D). Interestingly, we did not detect any ultrastructural differences in the SJs at 6 h post-mating, but we did uncover differences in SJ ultrastructure in different regions of the oviduct.

Based on their ultrastructure, two types of SJs, smooth and pleated, can be distinguished in *Drosophila *[26]. Smooth SJs are distinguished by the lack of visible septae and the appearance of electron dense material in the intercellular space, while pleated SJs are distinguished by the ladder-like appearance of septae. In the lateral oviducts and upper common oviduct, septa were not detected in the SJ, thus these SJs represent smooth SJs or an immature stage of pleated SJ (Figure 2A, 2A', 2A"). In contrast, a ladder-like arrangement of septae was often visible in the SJs of the lower common oviduct (Figure 2C'), thus these SJs can be classified as pleated. Unlike the smooth-like SJs of the upper oviduct, the pleated SJs of the lower oviduct are followed basally by spot type adherens junction (spot AJs) (Figure 2B, 2B'; additional file 1). Further analysis is necessary to determine if the SJs of the upper and lower oviduct represent different types or different developmental stages. Our findings demonstrate that the initial formation of SJs, as well as spot AJs in the lower oviduct, are mating-independent. This raises an interesting question. Why are SJ proteins such as Cora and Nrg up-regulated post-mating if SJs are formed prior to mating? It is possible that the increased expression of SJ proteins is associated with functional changes in polarized secretion post-mating. Recent studies have shown that SJs play an unexpected role in regulating the apical secretion of specialized extracellular matrix molecules in the trachea [27,28], and that these molecules are important regulators of lumen size.

### Mating modulates apical secretory activity in the oviduct

**Figure 3:**
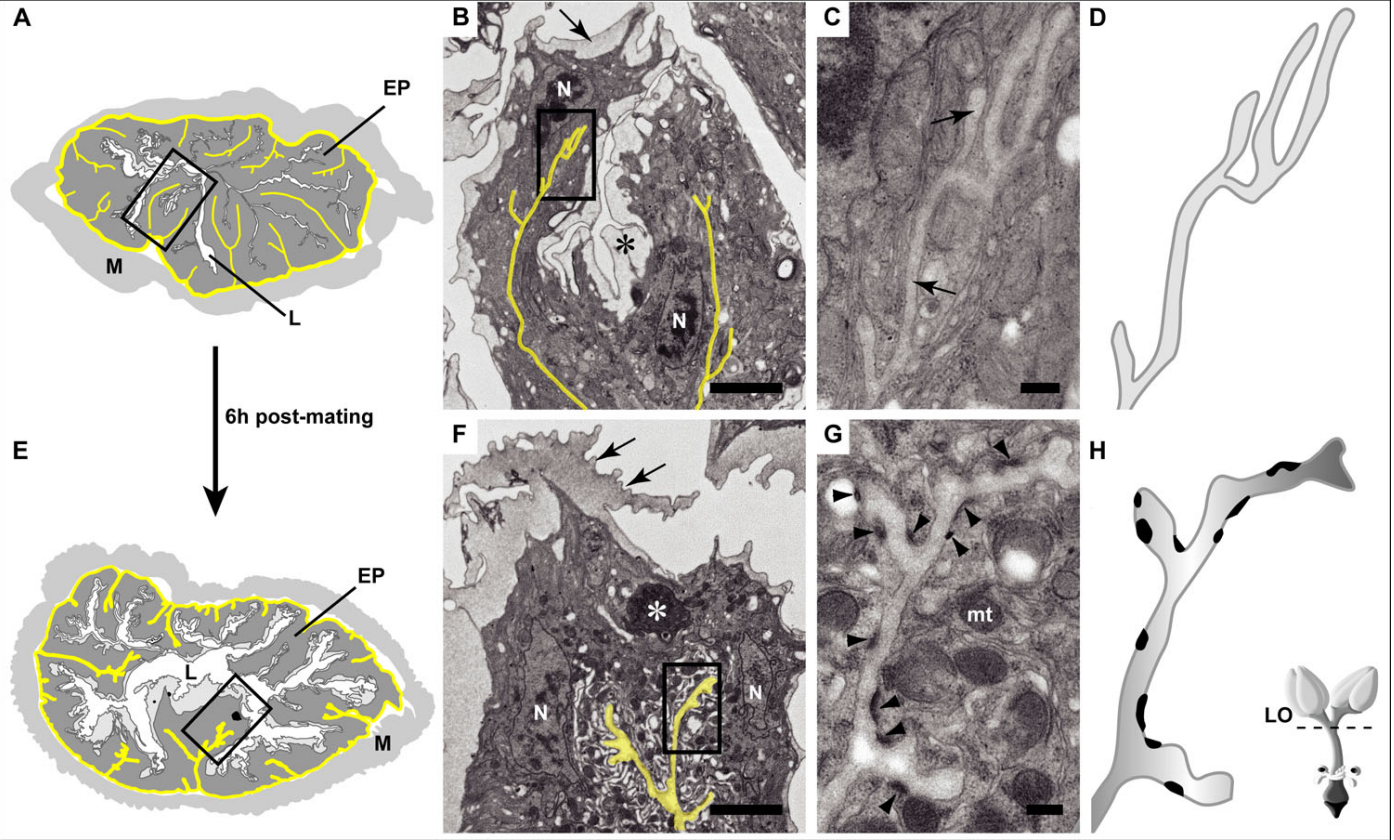
**Increased development of hemiadherens junctions (HAJs) and apical extracellular matrix (AECM) in upper oviduct after mating**. (A and E) Drawing of upper oviduct in cross section (traced from electron micrographs). Lumen (L) is barely visible in unmated (A) as compared to mated (E) oviduct. However, note the infolding of the basolateral membrane (outlined in yellow) in both unmated and mated. EP (epithelium); M (muscle). Electron micrographs of region outlined by box shown in B and F. (B and F) Basolateral infolding gives rise to an intercellular space with short branches (yellow). Apical surface is covered by an electron dense layer of matrix (black *) and thin layer of cuticle (arrow). The ruffled appearance of the cuticle (arrows in F) suggests an increase in surface area after mating. Note the presence of a granule (white*) in the cytoplasm. N (nuclei). Area outlined by box shown in C and G. Bar is 2 μm. (C and G) HAJs appear as electron dense patches underneath the plasma membrane. Note the absence of HAJs along the basolateral membrane (arrow) in unmated (C), and the abundance of HAJs (arrowheads), as well as mitochondria (mt) in mated (G). Bar is 200 nm. (D and H) Tracing of basolateral infolding highlights the appearance of HAJs after mating. Note also the increased width of the intercellular space in the mated oviduct (97.8 ± 12.4 μm versus 54.4 ± 11.7 μm). Schematic of the female reproductive tract showing the region of origin of the cross sections (dashed line, LO = lateral oviducts).

Given the presence of extensive SJs in the oviduct and the role of SJs in regulating apical secretion of extracellular matrix molecules in other epithelia (e.g. trachea), we asked if mating modulates apical secretion in the oviduct epithelia. Our ultrastructural analysis reveals that different regions of the oviduct display different apical membrane morphology (i.e microvilli or pleats) (see Figures 2 and additional file 2A and 2A), but all epithelia are covered by an electron dense apical extracellular matrix (AECM) and a thin layer of cuticle. We found that mating induces ultrastructural changes in the AECM and cuticle in both the upper and lower oviduct. In the upper oviduct of the unmated female, the AECM varies in thickness along the apical surface (Figure 3A). Some areas have little AECM, while other areas are covered by a distinct layer of AECM (~1–2 μm in thickness; Figure 3B). However, the AECM of mated females is more evenly distributed along the apical surface, (~2 μm in thickness; Figure 3E). Strikingly, the AECM and cuticle of mated females have a ruffled appearance, suggesting that the AECM and cuticle have increased in surface area (Figure 3F). Electron dense granules up to ~1.5 μm in diameter were occasionally observed in both the AECM and cell cytoplasm (Figure 3F). Although further analysis is needed to determine the role of these granules in the oviduct epithelia, it is possible that these granules participate in the secretion and deposition of the AECM. Taken together, our findings suggest that polarized secretion via the AECM, while ongoing in the upper oviduct of the unmated female is enhanced and/or modulated post-mating.

Post-mating changes in AECM ultrastructure are also observed in the lower common oviduct. However, unlike the AECM of the upper oviduct, the AECM of the lower oviduct is well developed prior to mating. In the lower oviduct of the unmated female, the AECM consists of an amorphous electron dense material and is unevenly distributed, forming a thick, bulbous layer above the plasma membrane in some regions (additional file 2). Matrix-like material is also observed in the lumen, but this material is more electron dense than the AECM (additional file 2A and 2A). In the mated female, the AECM is flattened against the plasma membrane and is uniformly distributed along the apical surface (additional file 2B and 2B). Matrix-like material was not observed in the center of the lumen, but small pools of very electron dense material were detected in the spaces between the epithelial folds (additional file 2C and 2C). This may explain why lumenal matrix was not detected at the light microscopic level in the mated female oviduct (see Figure 1E and 1F). Taken together, our observations suggest that the lower common oviduct is a site of active apical secretion in both mated and unmated females, and that matrix secretion, particularly in the lumen, is reduced post-mating. These findings raise the intriguing possibility that the AECM and lumenal matrix function as a plug in the lower oviduct, and that mating induces the breakdown of this plug.

### Mating induces changes in hemi-adherens junctions in upper oviduct

In addition to modulating secretion at the apical membrane, mating induces changes at the basolateral membrane. In many epithelia, one of the last steps of differentiation is the development of a layer of extracellular matrix (ECM) called the basal lamina that covers the apical and/or basal membranes and the concomitant development of hemi-adherens junctions (HAJs). HAJs connect the cell cytoskeleton with the ECM and are formed at virtually all cell surfaces that contact an ECM. HAJs can be distinguished at the ultrastructural level as a patch-like, electron dense undercoat of the plasma membrane that opposes the basal lamina ([26]; Figure 3G, 3H). In the *Drosophila *embryo, the HAJs and basal lamina are formed at the same time [26]. Because the basal lamina is established at a time when the majority of extracellular matrix molecules are actively secreted [26,29], this suggests that the formation of HAJs is tightly coordinated with the secretion of the ECM.

One of the most striking post-mating changes observed in the oviduct epithelia was the appearance of numerous HAJs along the basolateral membrane in the upper oviduct (Figure 3). The importance of HAJs, particularly in the upper oviduct, is underscored by the extensive infolding of the basolateral membrane that is observed in both unmated and mated females (Figure 3A and 3E). The infolded membrane gives rise to a highly branched intercellular space that is filled with an ECM (Figure 3C and 3G). This ECM is contiguous with the basal lamina that surrounds the epithelia. Few HAJs were observed in the upper oviduct of the unmated female, and these were largely restricted to the basal membrane, and not observed along the basolateral infolding (Figure 3C). In contrast, numerous HAJs appear along the basolateral infolding post-mating in this region of the oviduct (Figure 3F–3H). In addition, the intercellular space appears wider post-mating (Figure 3C–3D and 3G–3H), suggesting that mating induces increased secretion and/or deposition of the ECM in this cellular compartment and brings the ECM to a threshold concentration that can support the development of HAJs. HAJs were also detected along the basal membrane, but they were not detected along the apical membrane even though this membrane was covered by an ECM. Interestingly, while the basolateral membrane forms very shallow folds in the lower oviduct (see additional file 2), HAJs were observed along this membrane in unmated reproductive tracts (data not shown), thus suggesting that the epithelia is more differentiated in this region of the oviduct, and that the differentiation of the upper and lower oviduct may be under different control.

### Muscle differentiation is enhanced post-mating

**Figure 4:**
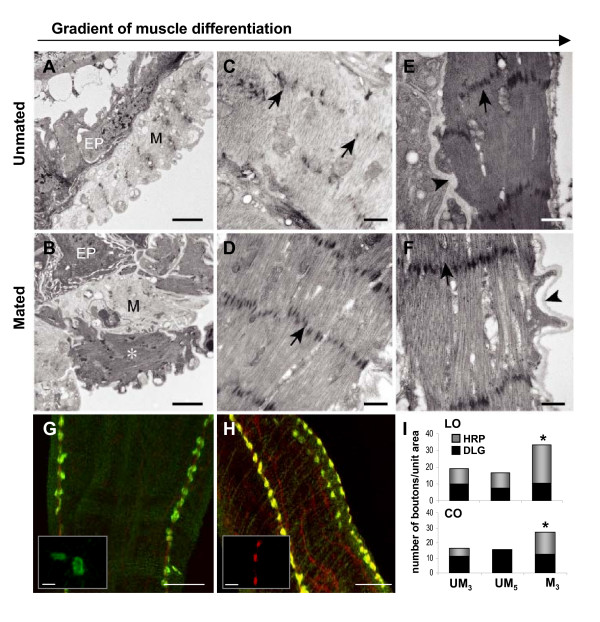
**Enhanced muscle differentiation and increased innervation observed post-mating**. (A-F) Ultrastructure of muscle in different regions of oviduct (unmated vs. mated). (A and B) Lateral oviducts: muscle fibers that vary in electron density are observed in this region. Light (M) and dark (*) muscle fibers may represent different stages of differentiation. Bar = 2 μm. Epithelium (EP); (C and D) Upper common oviduct: Z-bodies (arrows) are more organized in mated (D) than unmated muscles (C). Bar = 0.5 μm. (E and F) Lower oviducts in both unmated and mated: muscle fibers in the lower oviduct are more electron dense than muscle fibers in upper oviduct. An increased density of z-bodies (arrows) and myofilaments that run between the z-bodies was observed. Muscle fibers are surrounded by a well developed basal lamina (arrowheads). Bar = 0.5 μm. (G and H) Confocal images of immunolabeled type I and II boutons innervating the oviduct. Common oviducts of 3-day-old unmated (UM_3_) (G) and mated females (M_3_) (H), were stained with anti-DLG (green) and anti-HRP (red); Bar = 20 μm. Insets show high magnification of labeled type I (green) and II (red) boutons; bar = 5 μm. (I) Quantitation of number of immunolabeled boutons per unit area in lateral oviducts (LO) and common oviduct (CO). Anti-HRP labels both type I & II boutons, whereas anti-DLG only labels type I boutons. We found a significant increase in the number of HRP labeled boutons (lateral oviducts, *p *< 2.53E-05; common oviduct, *p *< 0.005) but not in the number of DLG labeled boutons, indicating that mating induces an increase in type II innervation. As a control, we also included 5-day-old unmated females (UM_5_) which showed the same innervation pattern as in UM_3 _oviducts. For each treatment oviducts were obtained from 3 biological replicate; in each replicate for each treatment n = 10 oviducts. See also additional file 3.

While we uncovered post-mating changes in the oviduct epithelia that might facilitate its transition to a high egg-laying state, this transition may also be mediated by changes in oviduct muscle properties and/or activity. The oviduct is lined by circular muscle fibers with supercontractile characteristics [30]. Our previous studies showed that *mef2 *and *mlp84B *genes that regulate muscle differentiation, are expressed and increased post-mating in the oviduct, as well as in the sperm storage regions of the reproductive tract [22,23]. This suggests that mating induces muscle differentiation in the reproductive tract. Muscle differentiation is characterized by the assembly of myofilaments into bundles called myofibrils. As muscles differentiate, myofibrils and z-bodies appear simultaneously, and increase in number until the cytoplasm is filled with myofibrils [31]. Like epithelia, one of the last steps of muscle differentiation is the secretion of a basal lamina that surrounds the muscle fiber. To determine if mating induces structural changes in muscles (such as increased myofibrils) we examined the ultrastructure of muscle fibers in the upper and lower parts of the oviduct. Our analysis revealed that, in both unmated and mated reproductive tracts, the muscles of the lower oviduct are highly differentiated as evidenced by the high density of myofibrils, well developed and aligned z-bodies, and secretion of a thick, electron dense basal lamina (Figure 4E and 4F). In contrast, muscle fibers in the lateral oviducts and upper common oviduct appear less differentiated than muscles in the lower common oviduct, as evidenced by the lesser density of myofibrils and z-bodies, and little or no basal lamina (Figure 4A and 4B). Moreover, the muscles of the upper oviduct appear more differentiated in the mated than in unmated reproductive tracts (Figure 4A–4D). Interestingly, we observed neighboring muscle fibers in different states of differentiation in the lateral oviducts in both unmated and mated reproductive tracts, but not elsewhere in the oviduct (Figure 4B). These results suggest that mating enhances the rate of muscle differentiation in the upper oviduct, and that muscle differentiation is delayed in the upper as compared to the lower oviduct. The increased muscle differentiation in the upper oviduct is not dramatic and likely reflects the short post-mating period examined in this study. The delayed differentiation of the upper oviduct muscles resembles the delay in the onset of development between the adult thoracic muscles and abdominal muscles during metamorphosis [32]. Since the ovaries and the other parts of the reproductive tract are known to have different segmental origins [33], we hypothesize that different parts of the oviduct develop at different rates or begin development at different times.

### Increased innervation in the oviduct post-mating

Nerve-muscle interactions play an important role in regulating adult muscle development and refining the final pattern of innervation [34,35]. Given that oviduct muscle differentiation is enhanced post-mating, we predicted that mating either directly or indirectly induces changes in innervation. To address this prediction, we quantified the number of nerve terminals or boutons innervating the lateral oviducts and common oviduct in unmated and mated reproductive tracts. Studies of oviduct innervation in *Drosophila *reveal that the fly's oviduct receives aminergic, peptidergic and glutamatergic input [30,36-39]. In both larval and adult *Drosophila*, different types of boutons are formed by neurons that express different neurotransmitters and modulators [40-42]. By similarity to the boutons described at the larval and adult neuromuscular junction, Middleton et al. [30] report that the fly's oviduct is innervated by glutamatergic type I boutons and tyraminergic/octopaminergic type II boutons. Rodgriguez-Valentin et al. [43] further report that the oviduct type II boutons co-express octopamine and glutamate. The neurons that give rise to the type I innervation have not been identified. However, it is well established that type II innervation arises from octopaminergic neurons located in the abdominal ganglion [30,43]. In addition, it has been shown that some or all of these neurons express a GAL4 insertion line for the *bullwinkle *(*bwk*) gene [43]. *Bwk *encodes a HMG-box containing putative transcription factor [44]. To determine if mating induces any changes in the number of type I and II boutons innervating the oviduct muscles, we used the pan-neural marker, anti-HRP to label all oviduct boutons in unmated and mated females. To distinguish between type I and II boutons we used an antibody against the Disc Large (DLG) protein [45]. Type I boutons were identified by their DLG postsynaptic staining and large size (> 8 μm in diameter) (Figure 4G), while type II boutons were distinguished by their absence of DLG staining and smaller size (< 2 μm) (Figure 4H). We find that type I and II boutons innervate the lateral oviducts and common oviduct, and that the type I innervation is restricted to a few axons that run parallel to the length of the oviduct, while the type II innervation is more widespread. We quantified the number of boutons in the lateral oviducts and common oviduct and observed a 74% increase in bouton number in the lateral oviduct and a 66% increase in the common oviduct post-mating (Figure 4I). More over, we observed no significant change in the number of type I boutons in the lateral oviduct and common oviduct. However, we detected a 1.5-fold increase in the number of type II boutons in the lateral oviduct and a 1.8-fold increase in type II innervation in the common oviduct. Dramatic increases in bouton growth are also observed during development. For example, a ten-fold increase in bouton number is observed at the neuromuscular junction during the larval period [46]. To determine if the increase in type II innervation was specific to mating or reflected normal growth in 3 day-old females, we quantified type I and II innervation in the oviducts of 5 day-old unmated females. We found no significant difference in type I and II innervation in unmated 3 day-old and 5-day-old females, indicating that mating, either directly or indirectly, induces a dramatic increase in type II innervation (Figure 4I). To determine if the post-mating increase in innervation is unique to the oviduct, we asked if mating induces a global change in innervation. We quantified bouton number in the adult ventral midline muscles of the 5^th ^abdominal segment. These muscles are innervated by boutons that increase in number during metamorphosis [47]. No significant difference in bouton number was detected at these muscles in unmated and mated females (additional file 3). Though further analysis is needed, this suggests that the post-mating increase in innervation is oviduct-specific. Because the type II boutons are octopaminergic, the increased type II innervation may result in increased octopamine (OA) release in the oviduct. In support of this possibility, we have preliminary evidence that OA is released in the oviduct post-mating (Heifetz and Wolfner, in preparation). Studies in locust and *Drosophila *demonstrate that OA inhibits oviduct contraction, while glutamate activates oviduct contraction [30,43,48]. In *Drosophila*, electrical stimulation of the posterior abdominal nerve gives rise to a series of muscle contractions in the oviduct followed by a period of muscle fatigue or relaxation [43]. This pattern of muscle contraction and relaxation may facilitate the proper movement of the egg through the oviduct. In their study of *bwk *expressing neurons that innervate the oviduct, Rodriguez-Valentin et al. [43] show that OA and glutamate interact to produce the pattern of oviduct contraction and relaxation described above. It is therefore possible that the post-mating increase in type II innervation in the oviduct plays an important role in the increased rate of ovulation and egg-laying observed post-mating.

### Female mating history affects the enrichment of cytosekeletal proteins in the oviduct

**Figure 5:**
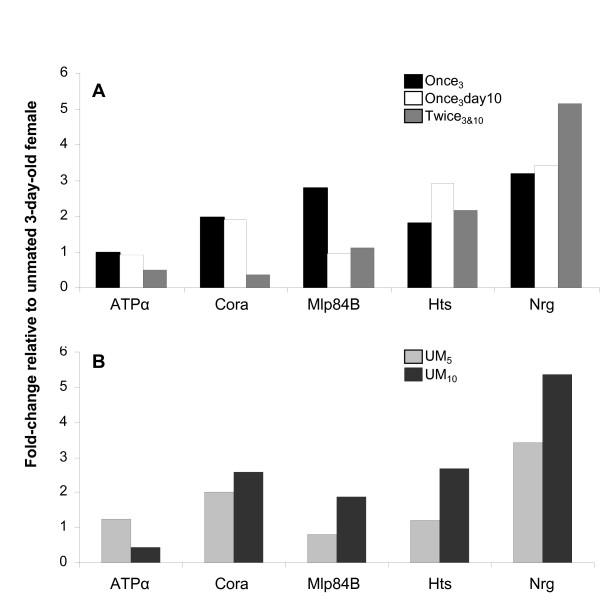
**Mating induces changes in the level of cytoskeletal proteins in the oviduct**. (A) The abundance of cytoskeletal proteins in oviducts dissected at different times post-mating and following different mating regimes was analyzed by Western blot. We calculated the abundance of each protein in mated females (described below) relative to the level in 3-day-old unmated females. Three different mating regimes were examined: (i) 3-day-old females that mated once at 3 days of age (once_3_), (ii) 10-day-old females that mated once at 3 days of age (Once_3 _day 10), and (iii) females that mated twice, first at 3 days and again at 10 days of age (Twice_3&10_). (B) We also calculated the abundance of each protein in the oviducts of: (i) 5-day-old unmated females relative to its level in oviducts of UM_3 _(UM_5_); (ii) 10-day-old unmated females relative to its level in oviducts of UM_3 _(UM_10_). For each treatment oviducts were obtained from 3–4 biological replicate; in each replicate for each treatment n = 60 oviducts/lane.

To gain insights into the role of cytoskeletal protein enrichment ([23]; additional file 4) in mediating the morphological changes detected in this study, we examined the effect of different mating regimes on cytoskeletal protein abundance. We focused on a subset of mating-responsive cytoskeletal proteins with well established roles in the differentiation of muscle and epithelia. These include: (i) Mlp84B which regulates the late differentiation pathway of muscle [49]; (ii) Cora and Nrg which are required for the formation of septate junctions in epithelia [50], and (iii) Hu-li tai shao (Hts), also known as adducin-like protein, which functions in assembly of the cytoskeletal network. Na^+ ^pump α subunit (ATPα), another protein associated with septate junctions in epithelia, is not a mating-responsive protein and was used as a control. Using western blots, we first determined the abundance of the cytoskeletal proteins in oviducts of 3-day-old unmated and mated females at 6 hrs post-mating. We confirmed the proteomic results of Kalpenikov et al. [23] and found that mating increases the abundance of all proteins, except ATPα in mated oviducts relative to their abundance in unmated oviducts (Figure 5A).

To determine whether the increased abundance of mating-responsive proteins persists for longer times post-mating, we examined oviducts of 10-day-old females that mated once at 3 days of age, and calculated the abundance of the mating-responsive proteins relative to their level in oviducts of 3-day-old unmated females. We found no change or a slight increase in the relative abundance of all cytoskeletal proteins except Mlp84B at 7 days post-mating (Figure 5A). Strikingly, the level of Mlp84B declines by 7 days post-mating to the level observed prior to mating. Thus Mlp84B levels rise and fall after mating, while the epithelial-related proteins rapidly rise and are maintained at a high level after mating. This raises the possibility that a second mating might trigger an increase in Mlp84B protein expression as observed in 3-day-old females at 6 h post-mating. To test this possibility, females were mated twice (once at day 3, and once on day 10 of age), and their oviducts were examined at 6 hrs after the second mating. We calculated the abundance of the cytoskeletal proteins in the twice mated oviducts relative to their abundance in oviducts of 3-day-old unmated females. Our results show that a second mating has little or no effect on Mlp84B abundance. Thus, Mlp84B may represent a class of mating-responsive proteins that is only needed after the first mating. Interestingly, the effect of the second mating on the epithelial-related mating-responsive proteins appears to be different for each protein. While Cora levels drop to the level observed in 3-day-old unmated females, Nrg and Hts are maintained at a high level.

To determine if the changes in cytoskeletal protein abundance are mating-dependent we measured their abundance in the oviducts of unmated 5- and 10-day-old females. We calculated their abundance relative to their level in oviducts of unmated 3-day-old females (Figure 5B). We rationalized that if the change in cytoskeletal protein abundance is mating-dependent we will not see similar changes in unmated females. We observed a slow increase in the relative abundance of all mating-responsive proteins with time post-eclosion (Figure 5B). Because unmated females lay more eggs as they age (see additional file 5C) one possible interpretation of the increased level of cytoskeletal proteins in unmated females is that these proteins are associated with an intrinsic program for oviduct maturation and that mating accelerates this process to maximize egg-laying efficacy. Alternatively, it is possible that the slow increase in protein abundance observed in unmated females is due to the passage of eggs through the oviduct.

Taken together, our results suggest that mating is essential to fine-tune the levels of the mating-responsive proteins examined in this study. Because the changes in cytoskeletal protein abundance are different in unmated and mated females, this suggests that the post-mating changes are mating-dependent. Furthermore, we suggest that these post-mating changes are linked to changes in oviduct function.

### Early or prior mating increases fecundity

**Figure 6:**
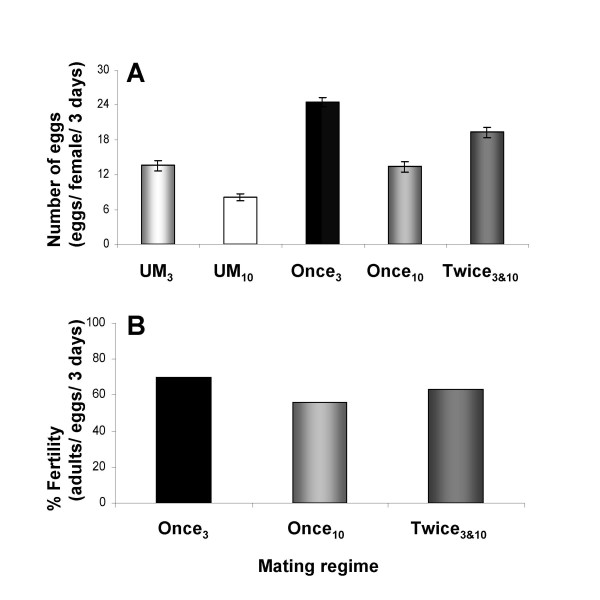
**Early or prior mating increases female fecundity**. Females were mated once at 3 days post-eclosion (Once_3_) or at 10 days post-eclosion (Once_10_) or twice at 3 and 10 days post-eclosion (Twice_3&10_). Eggs were counted at four time points: 6 hr, 1, 2 and 3 days post-mating. (A) Represents the average number of eggs laid by the female during the three days examined. Fertility was calculated as the average number of adult eclosed per laid eggs per female per day. (B) Represents the percentage of female fertility during the three days examined. We examined three independent biological replicates for each mating regime; n = 40–163 females for each replicate. See also additional file 5.

In *Drosophila*, female fecundity decreases with age [51-54]. It has been proposed that this decrease is due, in part, to the loss of germline and somatic stem cells [55]. Since the expression of the oviduct cytoskeletal proteins examined in this study change with age and mating experience, the state of the oviduct may also play a role in fecundity. To separate the effects of age and mating, we measured the fecundity of females that mated twice, first at 3 days post-eclosion and again at 10 days, and compared that to the fecundity of females that mated once at 3 days and females that mated once at 10 days. Fecundity was measured as the number of eggs laid per day per female during the first three days after mating. Once-mated 3-day-old females laid nearly twice as many eggs as once-mated 10-day-old females during the three days examined (24.5 ± 0.7 versus 13.3 ± 0.9, *p *< 0.0001). Twice-mated 10-day-old females also laid about 50% more eggs than once-mated females of the same age (19.3 ± 0.9 versus 13.3 ± 0.9, *p *< 0. 0001), but about 20% fewer than laid by once-mated 3-day-old females during the three days examined (19.3 ± 0.9 versus 24.5 ± 0.7, *p *< 0.0001) (Figure 6A, see also additional file 5A and 5B). Thus, the difference in fecundity between once-mated 10-day-old and once-mated 3-day-old females is not a result of age alone. Rather the main determinant of fecundity at 10 days is whether there had been a prior mating at 3 days. We also calculated the fertility (number of adults eclosed) of once- and twice-mated females. Once-mated 3-day-old females are more fertile than once-mated 10-day-old females (69.5 ± 1.6% versus 56.1 ± 3.0%, *p *< 0.0001) and slightly more fertile than twice-mated 10-day-old females (69.5 ± 1.6% versus 62.7 ± 2.4%, *p *< 0.015) (Figure 6B, see also additional file 5D). Because there is no significant difference in fertility between once-mated and twice-mated 10-day-old females, this suggests that (1) a prior mating has no significant effect on the fertility of 10-day-old mated females and (2) fertility decreases with age. One intriguing interpretation of these results is that an early mating increases fecundity which partially compensates for the age-related decrease in fertility. Thus, cytoskeletal mating-responsive protein changes may be associated with structural changes in the oviduct that result in increased fecundity. This may counteract the effects of decreased fertility on the reproductive output.

## Discussion

Despite the use of *Drosophila *as a model system for organ-level biology and the emerging parallels between mammalian and *Drosophila *reproductive biology [56], this is the first integrative tissue-wide study of post-mating changes in the *Drosophila *oviduct. Our results provide several lines of evidence at the molecular, morphological and physiological levels suggesting that mating induces tissue-wide differentiation in the oviduct. Moreover, we identify ultrastructural changes in the mated oviduct that are consistent with the roles that some of the mating-responsive proteins examined in this study (e.g. Mlp84B, Cora, Nrg) are reported to play in muscle and epithelial differentiation elsewhere. For example, the increased abundance of Mlp84B, a major regulator of the late differentiation pathway of muscle [49] is consistent with the increased muscle differentiation in the upper oviduct post-mating (Figure 4A–4F). Similarly, the increased abundance of Cora and Nrg, molecules that are essential for SJ development and function [50] is consistent with the observation that SJs in the upper oviduct are immature and/or that mating induces changes in the apical extracellular matrix whose secretion may be regulated, in part, by SJs as occurs in the trachea [27,28]. Other post-mating changes that indicate that mating induces tissue-wide differentiation include increased HAJs along the basolateral membrane and increased innervation.

Analysis of protein abundance following different mating regimes (unmated, once-mated, twice-mated) gave us further insights into the possible roles that mating-responsive proteins play in the oviduct. For example, Mlp84B is only responsive to the first mating, while the epithelial proteins examined (Cora, Nrg and Hts) are responsive to the first and second mating. Furthermore, the response to the second mating is different from the response to the first mating. Taken together, these results suggest that Mlp84B is required for the final maturation of the oviduct, while the epithelial proteins examined are required for both the maturation and maintenance of the oviduct at a high functional state. Moreover, the post-mating pattern of Mlp84B supports the idea that the first mating induces the final maturation of the oviduct. The rise and fall of Mlp84B abundance after the first mating (Figure 5) parallels the expression pattern of Mlp84B during development where peaks in *Mlp84B *transcription occur during periods of embryogenesis and metamorphosis when muscle is differentiating [49].

Using different mating regimes we tested whether the first/early mating is essential for maintenance of high reproductive output in the second mating. Our results suggest that mating at an early age is essential to achieve maximum reproductive output (i.e. high fecundity and fertility). Since the first mating increases reproductive output (evidence from our mating regime experiments), it is likely that the ultrastructural changes detected in mated 3-day-old females lead to a highly functional oviduct.

**Figure 7:**
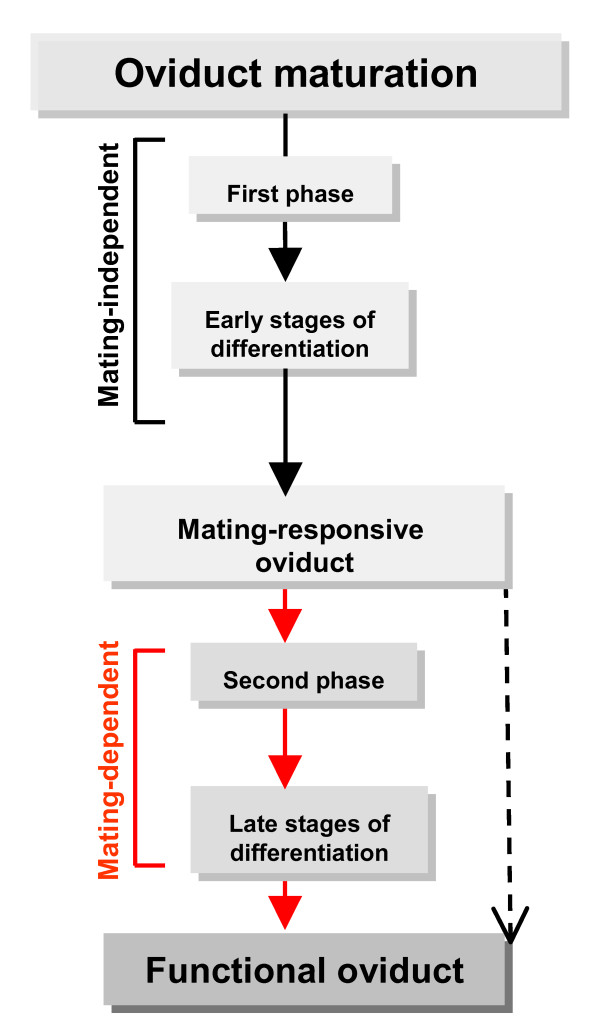
**Suggested model for oviduct maturation programs post-eclosion**. Shown is a flow chart suggesting themes in the last stage of oviduct maturation (post-eclosion) that leads to a functional duct capable of supporting high egg-laying and fertilization rates. Dashed arrow represents mating-independent processes; red arrows represent mating-dependent processes.

We suggest that the final maturation of the oviduct includes a mating-dependent stage. We propose that during the first few days post-eclosion, the oviduct undergoes the first phase of differentiation, after which the oviduct is developmentally poised for a rapid response to an extrinsic cue (mating). Mating then triggers the second phase of maturation (tissue remodeling and modulation) which is essential for proper oviduct function (Figure 7). We further propose that the second phase of maturation consists of processes that are mating-independent and -dependent, and that both of these pathways are essential to produce a functional oviduct. For example, initial formation of SJs occurs prior to mating (mating-independent) while the increased apical secretion and development of HAJs are mating-dependent. The oviduct musculature is an example where both mating-independent and -dependent processes play a role. Muscles are highly differentiated in the lower oviduct prior to mating, while muscle differentiation is ongoing in the upper oviduct and increases after mating. Although the onset of muscle differentiation in both regions is mating-independent, the further differentiation of muscle in the upper oviduct is mating-dependent. Another possible interpretation of the role of mating on oviduct maturation is that mating accelerates and synchronizes processes that are essential for the functional maturation of the oviduct. It will therefore be interesting to examine the oviducts of older females to determine the status of oviduct maturation.

What is the benefit of mating-induced differentiation of the oviduct tissues? Unmated females are capable of laying eggs, albeit at a reduced rate as compared to mated females of the same age. One possible interpretation is that reproduction is energetically costly, thus delaying oviduct maturation until sperm is available is advantageous to the female. This may reflect the evolution of a mechanism to optimize reproductive capacity in early adulthood in short-lived animals.

In summary, we have identified events at the cellular, molecular and physiological levels that are part of an efficient and specific program for reproduction. *Drosophila *affords us the opportunity to uncover the signaling pathways that coordinate these events to produce a physiologically functional organ.

## Methods

### Flies

Wild-type Canton-S flies were used for the fecundity/fertility experiments and confocal analysis. Wild-type Canton-S5 [57] flies were used for electron microscopy. All flies were kept in a 12 hrs light/dark cycle at 23 ± 2°C. Upon eclosion, females and males were collected on ice and held separately until 3 (females and males) or 10 (females) days of age.

### Sample preparation

For all assays (unless described differently) unmated females were placed with 3-day-old unmated males and observed until mating initiated. At the end of mating, females were aspirated into fresh vials and held for 6 hrs. At 6 hrs after the start of mating, females were placed on ice for dissection. Previous molecular studies indicate changes in protein abundance at 3 hrs post-mating. We hypothesize that these molecular changes will translate into morphological changes in the next few hours. We chose to analyze the morphology of mated females at 6 h post-mating as opposed to later times post-mating because the changes observed at later times may be due, in part, to the high rate of eggs passing through the oviduct.

### Electron microscopy

Reproductive tracts were dissected in Schneider's *Drosophila *medium (Sigma) on ice and processed for electron microscopy as described in [42]. Tracts were flat-embedded between two sheets of Aclar (Electron Microscopy Sciences), which allowed us to image the entire tract at the light microscopic level prior to sectioning. Sections were cut on a Reichart Ultracut microtome. One-μm thick sections were stained with 1% toluidine blue, and viewed with a Zeiss Axoplan microscope. Ultrathin sections (~100 nm) were mounted on formvar grids, stained with lead citrate, and viewed with a Philips/FEI Morgagni 268 TEM at 80 kV. Our analysis is based on 4 unmated samples and 3 mated samples. Two unmated samples were cut in the longitudinal plane and two additional unmated samples were cut in the transverse plane, while one mated sample was cut in the longitudinal plane and two additional mated samples were cut in the transverse plane. For longitudinal sections, the entire tract was re-embedded and cut. For samples cut in the transverse plane, the flat-embedded reproductive tract was divided into three regions: (1) lateral oviducts and upper common oviducts, (2) middle common oviduct, and (3) lower common oviduct. Each region was re-embedded and sectioned. It is beyond the scope of this paper to describe all three regions, and our analysis focuses on the uppermost and lowermost regions.

### Immunocytochemistry

Reproductive tracts were dissected in Yamamoto's Ringer (10 mM MOPS; 80 mM NaCl; 10 mM KCL; 0.2 mM MgCl_2_; 0.1 mM CaCl_2_) with 5% (w/v) sucrose on ice, fixed in 4% paraphormaldehyde in PBS (phosphate-buffered saline; 0.85% NaCl, 1.4 mM KH_2 _PO_4_, 8 mM Na_2 _HPO_4_, pH 7.4) for 45 min and then washed in PBS. The reproductive tracts were then incubated in blocking solution (0.5% Triton x-100, 3% NGS, 0.1% BSA) for 2 hrs at room temperature. The following primary antibodies, reagents, and dilutions were used: Cy3-conjugated goat anti-HRP, 1:200 (Jackson Immunochemicals, West Grove, PA); mouse anti-Disc Large (DLG), 1:1000 (Developmental Hybridoma Bank), Alexa Fluor 488-phalloidin, 1:200 (Invitrogen, Molecular Probes, Scotland). Secondary antibodies were Alexa Flour 488-conjugated Goat anti-mouse, 1:200 and Alexa Flour 546-conjugated Goat anti-rabbit, 1:200 (Invitrogen, Molecular Probes, Scotland). Reproductive tracts were incubated with the different primary antibodies (diluted in PBS + 0.2% Triton x-100) for 2 hr at room temperature, washed with PBST, incubated with secondary antibodies for 2 hrs at room temperature and washed with PBS. Reproductive tracts of the different treatments were mounted with Antifade media [58] on a multi-well glass slide (Hendley-Essex, UK). For each treatment (unmated, mated) and antibody/reagent (HRP, DLG, phalloidin) a minimum of ten reproductive tracts from at least two independent biological replicates were prepared.

### Confocal microscopy

Reproductive tracts were viewed with a Zeiss 510 laser scanning confocal microscope using 20× and 60× objective with additional zooming. Optical sections from different focal plans of each reproductive tract region (lateral oviducts, common oviduct, uterus) were collected and projected as a reconstructed three-dimensional image using LSM image browser (version 3,5,0,376) software. Image collections were identical for each of the different reproductive tract regions analyzed.

### Quantitation of bouton number

To quantify the number of boutons in the lateral and common oviducts we used ImageJ software (1.37b, National Institutes of Health) to analyze confocal images of anti-HRP and anti-DLG labeled boutons in the oviduct. The number of boutons per unit area was quantified with the Particle Analysis Tool. Briefly, to differentiate between the boutons, the particle analysis tool requires the image to be a "binary" image (i.e., black or white), thus we first converted the images to gray scale. We then set a "threshold" range so that pixels in the image whose value lies in this range are converted to black; pixels with values outside this range are converted to white. We next defined a region of interest (ROI) within the oviduct to count particles (i.e. count boutons). This ROI was saved and served to measure the number of boutons per unit area in each treatment. For each oviduct we counted the number of anti-HRP and anti-DLG labeled boutons in two ROIs within the lateral oviducts and two ROIs in the common oviduct. One-way ANOVA (SPSS 15.0) was used to measure the difference in bouton number per unit area in different regions of the oviducts, in both unmated and mated females.

### Quantitation of cytoskeleton proteins

#### Sample preparation

To evaluate the effect of mating on the abundance of the cytoskeleton proteins tested, females were: (i) aged for 3 days, mated with 3-day-old unmated males and their oviducts were dissected after 6 hrs post-mating (Once_3_); (ii) aged for 3 days, mated with 3-day-old unmated males and their oviducts were dissected after 7 days (Once_3 day10_); (iii) aged for 3 days, mated first with 3-day-old unmated males and held singly for 7 days. At 10 days of age, female were mated again with 3-day-old unmated males. Oviducts were dissected at 6 hrs post-second mating (Twice_3&10_). We also examined 5-day-old and 10-day-old unmated females (UM_5_, UM_10 _respectively).

#### SDS polyacrylamide gel electrophoresis (SDS-PAGE) and Western blotting

For each mating regime, sixty oviducts were pooled and 30 μl of SDS-PAGE sample buffer was added as described in [59]. Samples were boiled, and then frozen at -20°C until loading. SDS-PAGE was performed on 12% polyacrylamide gels and western blotted as in [60]. Proteins were cross-linked to the filter. The following primary antibodies and dilutions were used: mouse anti-Neuroglian (kindly provided by M. Hortsch) 1:250; Guinea pig anti-Coracle (kindly provided by R.G. Fehon) 1:2500; rabbit anti-Mlp84B (kindly provided by M. Beckerle) 1:1000; mouse anti-hts (1B1, Developmental Studies Hybridoma Bank, DSHB) 1:75; mouse anti-Na, K-ATPase (α5, DSHB) 1:100. Secondary antibodies included: anti-Guinea pig IgG (peroxidase conjugated), anti-Rabbit IgG and anti-Mouse IgG (developed in goat, Sigma, Israel) 1:10,000. Proteins were visualized using an enhanced chemiluminescence (ECL) detection system (Amersham Piscataway, NJ).

#### Analysis

The developed film was scanned and the signal intensity (protein abundance) of each band was determined using ImageJ software (1.37d, National Institutes of Health). We evaluated protein abundance by measuring the mean gray value of a specific band and the background. The mean gray value of the background was then subtracted from that of the measured band. Relative protein abundance in mated oviduct vs. 3-day-old unmated oviduct was then calculated. Four independent biological replicates were prepared for each mating status. The reported abundance (see Figure 5) is the relative ratio (mated/unmated or unmated/unmated) of at least three replicates that showed the same trend.

### Examination of female reproductive output

#### Mating regimes

To evaluate the effect of mating on reproductive output females were treated as follows: (i) aged for 3 days and mated with 3-day-old unmated males (Once_3_); (ii) aged for 10 days and mated with 3-day-old unmated males (Once_10_); (iii) aged for 3 days, mated first with 3-day-old unmated males, held for 7 days and mated again with 3-day-old unmated males (Twice_3&10_). In all cases male and female pairs were observed to record mating initiation and termination.

#### Analysis

Following mating, females were aspirated into fresh vials, held singly and allowed to lay eggs for 6 hrs, then transferred daily (each 24 hrs) to fresh vials. The number of eggs laid and the number of eclosed adults were counted from vials created at 6 hrs, 1, 2 and 3 days post-mating. To ascertain the baseline of female egg-laying, we also included in our experiment unmated females that were kept in the same conditions as mated females. The number of eggs laid by unmated females was counted from vials created at 6 hrs, 1, 2 and 3 days after placing the females in the holding vials. In addition, we also recorded the pattern of unmated female egg-laying for 10 days.

To determine the effect of different mating regimes on female reproductive output (i.e. fecundity and fertility), we used One-way ANOVA (SPSS 15.0).

## Abbreviations

Nrg: Neuroglian; Cora: Coracle; Spec: α- and β-Spectrin; SJ: septate junction; SSJ: smooth septate junction; PSJ: pleated septate junction; ZA: zonal adherens junction; AJ: adherens junction; AECM: apical extracellular matrix; ECM: extracellular matrix; HAJ: hemi-adherens junction; SAJ: spot adherens junction; OA: Octopamine; Hts: Hu-li tai shao; ATP α: Na^+ ^pump α subunit; Mlp84B: Muscle LIM protein at 84B; DLG: Disc Large; HRP: horseradish peroxidas; UM: unmated; M: mated.

## Authors' contributions

AK and PKR contributed equally to this manuscript. AK, PKR and YH conceived and designed the project and analyzed the data. AK performed the confocal, Western blots and fertility assays. PKR and AK performed the light microscopy. PKR conducted the electron microscopy. AK, PKR and YH wrote the manuscript. RRH contributed to design of the study and revision of the manuscript. All authors participated in the discussion and approval of the final manuscript.

## Supplementary Material

Additional file 1 **Cellular junctions are established in the lower oviduct prior to mating**. (A) Junctional complex is comprised of an apical pleated septate junction (PSJ) and a basal spot adherens junction (SAJ). (B and C) Electron micrograph of junctional complex. Pleated septate junctions contains visible septae (arrowhead) and interdigitations (*). Spot adherens junction is distinguished by an electron dense undercoat of plasma membrane (arrow). Apical membrane covered by long microvilli (MV). Bar is 0.5 μm. Click here for file

Additional file 2 **Changes in AECM and lumenal matrix in lower oviduct**. Cross section of lower oviduct was traced from electron micrographs (top left, unmated; bottom left, mated). Color coded as follows: gray = muscle; orange = epithelial soma layer; yellow = AECM and microvilli; nuclei also indicated. Areas labeled A, B and C are shown in corresponding panel of electron micrographs. (A') thick layer of AECM in this region prior to mating. Luminal matrix also observed (arrowhead) (A") higher mag of luminal matrix (*). (B') AECM is flattened against the brush-like border of microvilli (thick arrow). (B") higher magnification of AECM and overlying cuticle (arrowheads). (C') lumenal matrix detected between epithelial folds (arrow). (C") higher magnification of lumenal matrix (*) and cuticle (arrowheads). N (nuclei); M (muscle); MV (microvilli). Click here for file

Additional file 3 **The abundance of boutons innervating the adult ventral abdominal muscles (segment V) is not changing post-mating**. (A) Abdominal muscles (green, stained with phalloidin) visualized in a dissected preparation of 3-day-old unmated female. Innervation of these muscles is visualized with anti-HRP (red). Ventral midline is indicated by dashed line. Abdominal segments 1–5 are shown. Boutons were counted in 5^th ^abdominal segment; (B) Graph shows number of boutons per unit area in unmated and mated females. Boutons were counted in 3-day-old unmated females (UM_3_) and mated females at 6 h post-mating (M_3_). The number of boutons decreased in mated females (M_3_) but the difference was not significant. Standard error shown for each treatment. Higher magnification shows strings of boutons (arrow) in UM_3 _(C) and in M_3 _(D). Click here for file

Additional file 4 **Table of mating-responsive oviduct cytoskeleton proteins.** Click here for file

Additional file 5 **Female fecundity is highest in once mated 3-day-old females**. Number of eggs laid by 3-day-old and 10-day-old unmated and mated females was examined. In addition, we also examined twice mated 10-day-old females (mated first at 3 days of age and mated second at 10 days of age). Number of eggs laid by unmated and mated females was counted in parallel. Four different times post-mating were examined: 6 h, 1 day (1 d), 2 days (2 d) and 3 days (3 d). (A) 3-day-old females: At all times examined except 3 d post-mating, once mated females (Once_3_) laid significantly more eggs than unmated females (UM_3_) of the same age (*p *< 0.005). Standard error (SE) is shown for each treatment at each time examined. (B) 10-day-old females: At all times examined except 6 hrs post-mating, once mated females (Once_10_) laid significantly more eggs than unmated females (UM_10_) of the same age (*p *< 0.005). At all times post-mating, 10-day-old twice mated females (Twice_3&10_) laid significantly more eggs than Once_10 _females (*p *< 0.014 at 6 hrs post-mating; *p *< 0.0001 at 1 d–3 d post-mating). SE is shown for each treatment at each time examined. (C) Egg-laying pattern of unmated females: Egg-laying was recorded from day 3 to day 10 post-eclosion. Females were kept singly in vials beginning at day 3. On each day eggs were counted and females were transferred to a new vial. Variation in egg-laying was observed from individual-to-individual. However, the number of eggs laid at day 3 was significantly different (*p *< 0.005) from the number of eggs laid at day 4. Thereafter the number of eggs laid by unmated female at each day examined was not significantly different from the number of eggs laid at the following day. SE is shown for each day. (D) Fertility was calculated at different times post-mating. Fertility is defined as the average number of adults that eclosed from the total number of eggs laid per female at each post-mating time examined. Overall, female fertility was not significantly affected by the different mating regimes at each time examined. In only one case did we observe a significant difference in fertility (*). At 2 d post-mating, fertility was higher in Twice_3&10_females as compared to Once_10 _females (*p *< 0.022). See also Figure 6. Click here for file
