# Supplementary figures and images for: Tissue remodeling: a mating-induced differentiation program for the Drosophila oviduct
Source: BMC Dev Biol. 2008 Dec 8;8:114. doi: 10.1186/1471-213X-8-114 (PMC2636784; doi:10.1186/1471-213X-8-114)

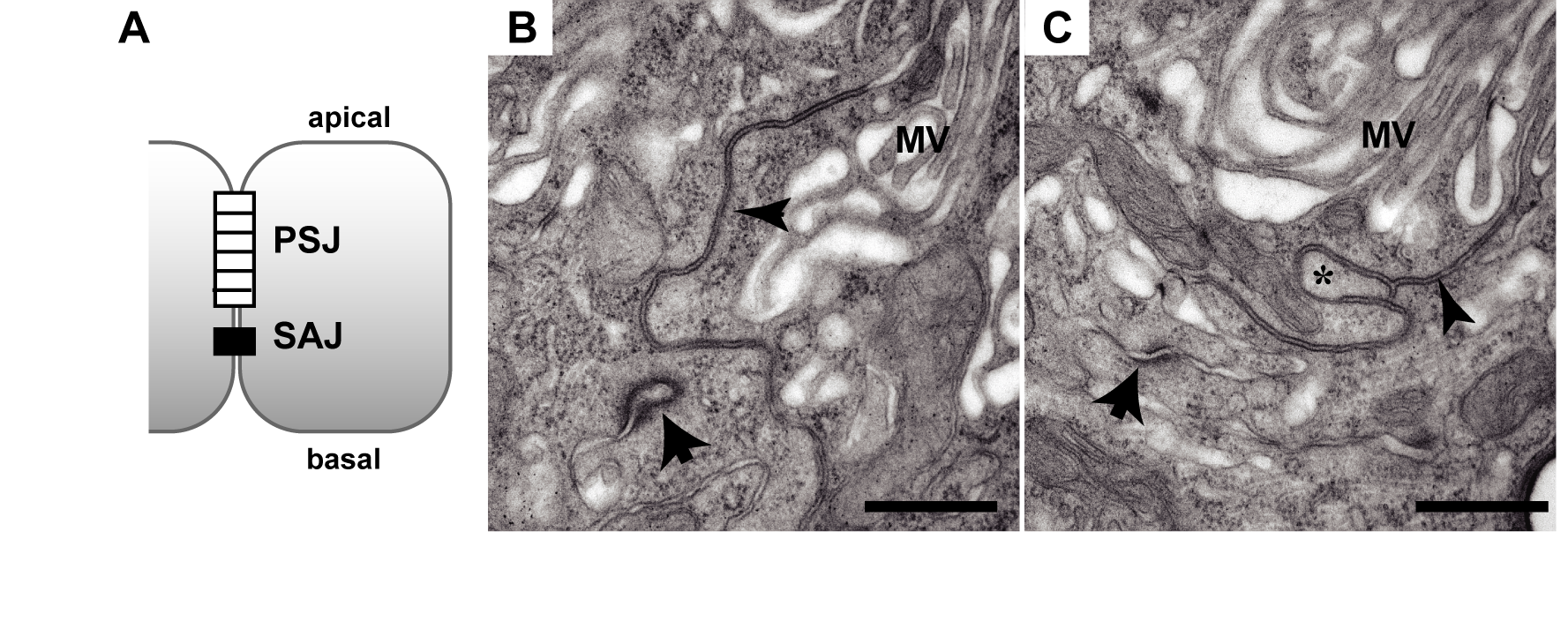

Supplement: Additional file 1 — Cellular junctions are established in the lower oviduct prior to mating. (A) Junctional complex is comprised of an apical pleated septate junction (PSJ) and a basal spot adherens junction (SAJ). (B and C) Electron micrograph of junctional complex. Pleated septate junctions contains visible septae (arrowhead) and interdigitations (*). Spot adherens junction is distinguished by an electron dense undercoat of plasma membrane (arrow). Apical membrane covered by long microvilli (MV). Bar is 0.5 μm. [file 1471-213X-8-114-S1.tiff]

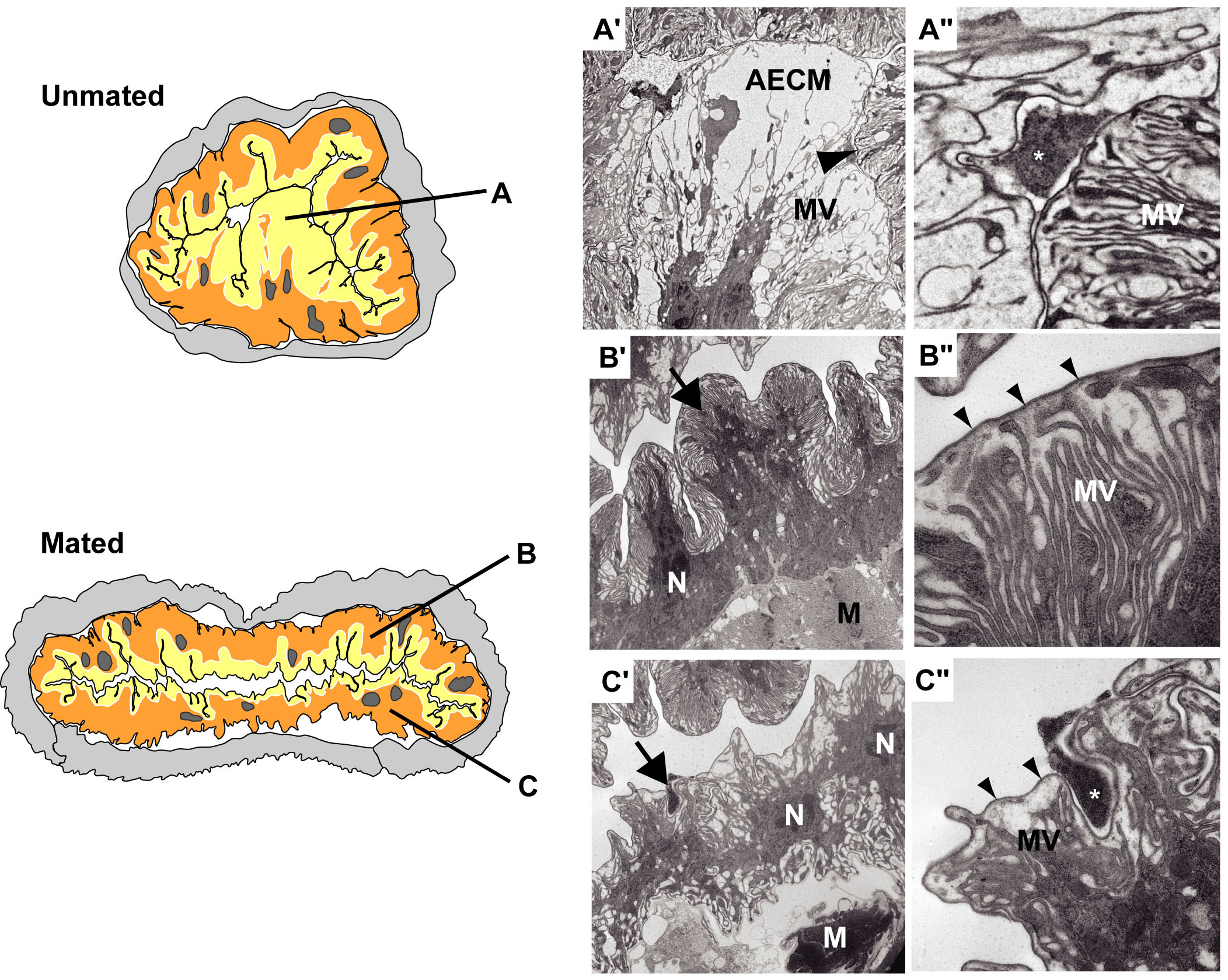

Supplement: Additional file 2 — Changes in AECM and lumenal matrix in lower oviduct. Cross section of lower oviduct was traced from electron micrographs (top left, unmated; bottom left, mated). Color coded as follows: gray = muscle; orange = epithelial soma layer; yellow = AECM and microvilli; nuclei also indicated. Areas labeled A, B and C are shown in corresponding panel of electron micrographs. (A') thick layer of AECM in this region prior to mating. Luminal matrix also observed (arrowhead) (A") higher mag of luminal matrix (*). (B') AECM is flattened against the brush-like border of microvilli (thick arrow). (B") higher magnification of AECM and overlying cuticle (arrowheads). (C') lumenal matrix detected between epithelial folds (arrow). (C") higher magnification of lumenal matrix (*) and cuticle (arrowheads). N (nuclei); M (muscle); MV (microvilli). [file 1471-213X-8-114-S2.jpeg]

## Slide 1
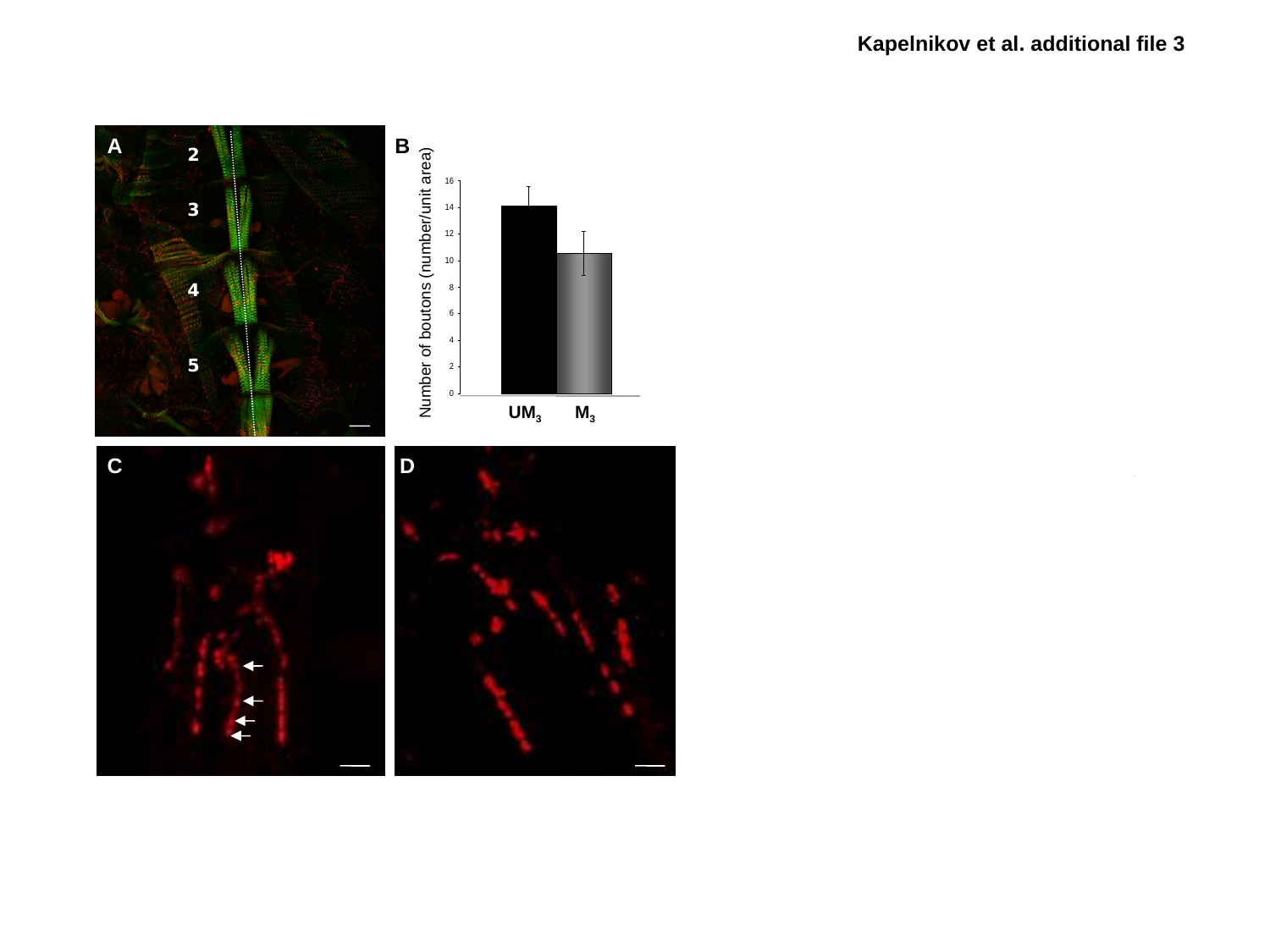

Kapelnikov et al. additional file 3
A
B
2
3
Number of boutons (number/unit area)
4
5
UM3
M3
C
D

Supplement: Additional file 3 — The abundance of boutons innervating the adult ventral abdominal muscles (segment V) is not changing post-mating. (A) Abdominal muscles (green, stained with phalloidin) visualized in a dissected preparation of 3-day-old unmated female. Innervation of these muscles is visualized with anti-HRP (red). Ventral midline is indicated by dashed line. Abdominal segments 1–5 are shown. Boutons were counted in 5th abdominal segment; (B) Graph shows number of boutons per unit area in unmated and mated females. Boutons were counted in 3-day-old unmated females (UM3) and mated females at 6 h post-mating (M3). The number of boutons decreased in mated females (M3) but the difference was not significant. Standard error shown for each treatment. Higher magnification shows strings of boutons (arrow) in UM3 (C) and in M3 (D). [file 1471-213X-8-114-S3.ppt]
