# Supplementary material for: Tissue remodeling: a mating-induced differentiation program for the Drosophila oviduct
Source: BMC Dev Biol. 2008 Dec 8;8:114. doi: 10.1186/1471-213X-8-114 (PMC2636784; doi:10.1186/1471-213X-8-114)
Supplement: Additional file 4 — Table of mating-responsive oviduct cytoskeleton proteins. [file 1471-213X-8-114-S4.pdf]

Table S1 - Mating-responsive oviduct cytoskeleton proteins

FBgn	Protein Name	Relative abundance* (M/UM)	Location in the cell	Molecular function
FBgn0002789	Mp20	0.50	contractile fiber	acting binding, calcium ion binding
FBgn0003149	Prm	2.05	contractile fiber	cytoskeletal protein binding
FBgn0002741	Mhc	1.56	Adherens junction/Cell matrix interaction	structural constituent of muscle
FBgn0014863	Mlp84B	2.75	Cytoplasm; Nucleus	protein binding; zinc ion binding
FBgn0005666	bt	6.25	cytoplasm	protein serine/threonine kinase activity; myosin light chain kinase activity
FBgn0004169	up	0.68	Adherens junction/Cell matrix interaction	tropomyosin binding
FBgn0002921	ATPα[#]	1.62	septate junction	ATP binding
FBgn0002527	LanB1	2.50	Basal lamina; Basement membrane	tissue development
FBgn0001219	Hsc70-4	2.56	Adherens junction/Cell matrix interaction	ATPase activity
FBgn0001402	trol (perlecan)	2.63	Extracellular matrix	structural molecule activity
FBgn0016724	RfaBp	2.88	Extracellular matrix	fatty acid binding
FBgn0010397	LamC	3.00	Extracellular matrix	structural constituent of cytoskeleton
FBgn0002968	Nrg	3.50	septate junction	calcium ion binding
FBgn0000667	alpha actinin	4.00	Adherens junction/Cell matrix interaction	actin filament binding
FBgn0003471	beta spec	4.00	Marginal Zone/Septate junction	cytoskeletal protein binding
FBgn0004873	hts	5.50	Marginal Zone/Septate junction	actin binding
FBgn0010434	Cora	5.83	septate junction	cytoskeletal protein binding
FBgn0003470	alpha-spec	6.00	Marginal Zone/Septate junction	cytoskeletal protein binding

Relative abundance - abundance of the protein in oviduct of mated female (M) relative to its abundance in oviduct of unmated (UM) female

*** -** mating-responsive proteins identified by MudPIT in oviducts of unamted and mated females (3hrs post-mating; see also [23])

[#] - non-mating responsive oviduct proteins
