# Supplementary material for: Tissue remodeling: a mating-induced differentiation program for the Drosophila oviduct
Source: BMC Dev Biol. 2008 Dec 8;8:114. doi: 10.1186/1471-213X-8-114 (PMC2636784; doi:10.1186/1471-213X-8-114)
Supplement: Additional file 5 — Female fecundity is highest in once mated 3-day-old females. Number of eggs laid by 3-day-old and 10-day-old unmated and mated females was examined. In addition, we also examined twice mated 10-day-old females (mated first at 3 days of age and mated second at 10 days of age). Number of eggs laid by unmated and mated females was counted in parallel. Four different times post-mating were examined: 6 h, 1 day (1 d), 2 days (2 d) and 3 days (3 d). (A) 3-day-old females: At all times examined except 3 d post-mating, once mated females (Once3) laid significantly more eggs than unmated females (UM3) of the same age (p < 0.005). Standard error (SE) is shown for each treatment at each time examined. (B) 10-day-old females: At all times examined except 6 hrs post-mating, once mated females (Once10) laid significantly more eggs than unmated females (UM10) of the same age (p < 0.005). At all times post-mating, 10-day-old twice mated females (Twice3&10) laid significantly more eggs than Once10 females (p < 0.014 at 6 hrs post-mating; p < 0.0001 at 1 d–3 d post-mating). SE is shown for each treatment at each time examined. (C) Egg-laying pattern of unmated females: Egg-laying was recorded from day 3 to day 10 post-eclosion. Females were kept singly in vials beginning at day 3. On each day eggs were counted and females were transferred to a new vial. Variation in egg-laying was observed from individual-to-individual. However, the number of eggs laid at day 3 was significantly different (p < 0.005) from the number of eggs laid at day 4. Thereafter the number of eggs laid by unmated female at each day examined was not significantly different from the number of eggs laid at the following day. SE is shown for each day. (D) Fertility was calculated at different times post-mating. Fertility is defined as the average number of adults that eclosed from the total number of eggs laid per female at each post-mating time examined. Overall, female fertility was not significantly affected by th [file 1471-213X-8-114-S5.ppt]

## Slide 1
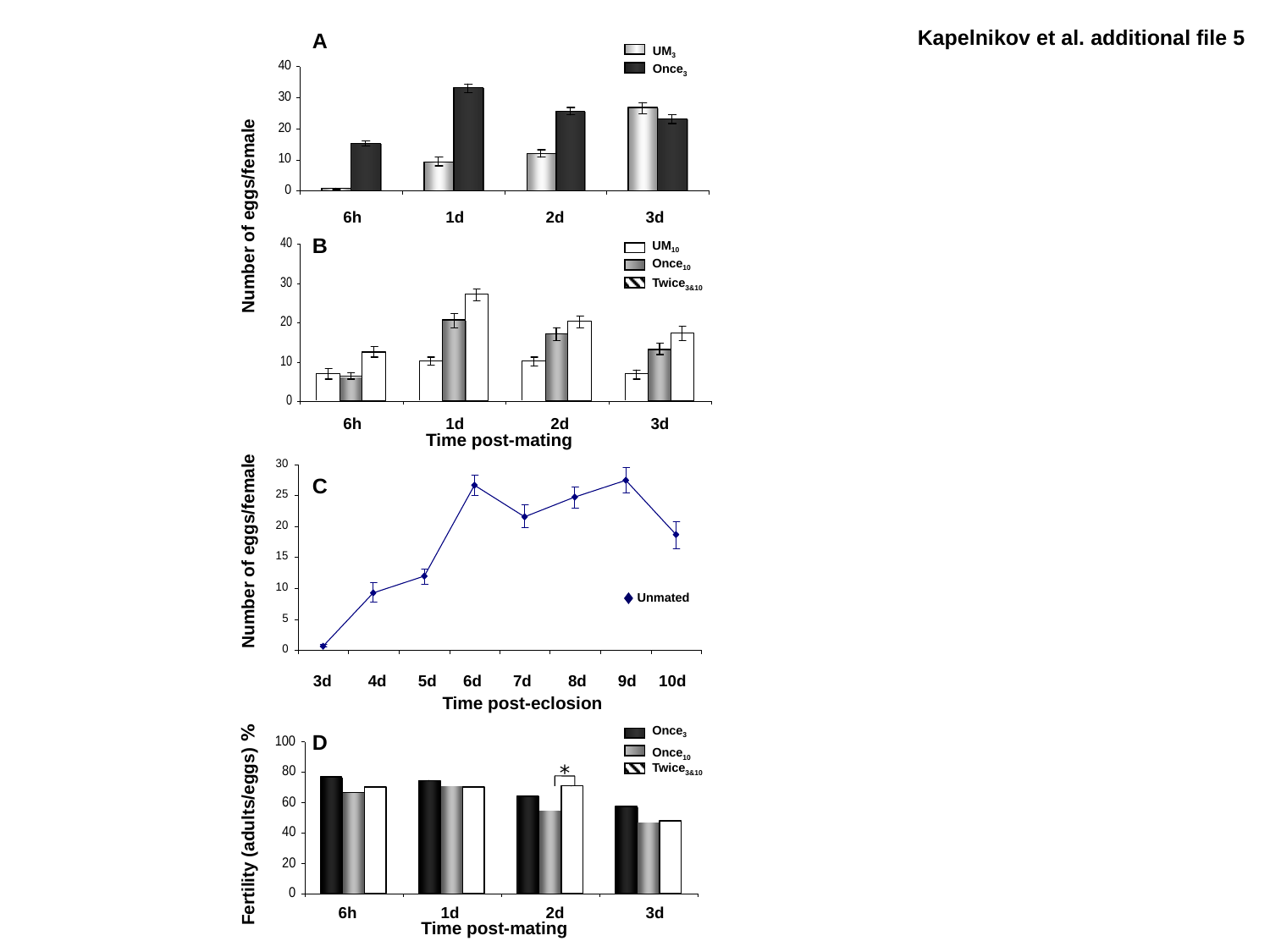

Kapelnikov et al. additional file 5
A
UM3
Once3
Number of eggs/female
6h
1d
2d
3d
B
UM10
Once10
Twice3&10
6h
1d
2d
3d
Time post-mating
C
Number of eggs/female
Unmated
3d
4d
5d
6d
7d
8d
9d
10d
Time post-eclosion
Once3
Once10
Twice3&10
D
*
% Fertility (adults/eggs)
6h
1d
2d
3d
Time post-mating
